# FDG PET/CT evaluation of pathologically proven pulmonary lesions in an area of high endemic granulomatous disease

**DOI:** 10.1007/s12149-013-0695-7

**Published:** 2013-02-12

**Authors:** Ronnie Sebro, Carina Mari Aparici, Miguel Hernandez-Pampaloni

**Affiliations:** 1 Department of Radiology and Biomedical Imaging, University of California, San Francisco, 505 Parnassus Avenue, M-391, San Francisco, CA 94143 USA; 2 Department of Radiology and Biomedical Imaging, University of California, San Francisco, 513 Parnassus Avenue, S965, San Francisco, CA 94143 USA

**Keywords:** PET/CT, Solitary pulmonary nodule, Granulomatous disease, Emphysema

## Abstract

**Purpose:**

The goal of this study is to assess how reliable the threshold maximum standardized uptake value (maxSUV) of 2.5 on positron emission tomography–computed tomography (PET/CT) is for evaluation of solitary pulmonary lesions in an area of endemic granulomatous disease and to consider other imaging findings that may increase the accuracy of PET/CT.

**Materials and methods:**

The staging PET/CT of 72 subjects with solitary pulmonary lesions (nodules (less than 3 cm) or masses (greater than 3 cm)) were retrospectively reviewed. Pathology proven diagnosis from tissue samples was used as the gold standard. Logistic regression was used to assess whether the subject’s age, maxSUV, size of lesion, presence of emphysema, or evidence of granulomatous disease was predictive of malignancy.

**Results:**

Malignant lesions were identified in 84.7 % (61/72) of the 72 subjects. A threshold maxSUV of 2.5 had a sensitivity of 95.1 % (58/61), specificity of 45.5 % (5/11), positive predictive value of 90.6 % (58/64), negative predictive value of 62.5 % (5/8) and an accuracy of 87.5 % (63/72). The false negative rate was 4.9 %, and the false positive rate was 54.5 %. All 3 false negatives were less than or equal to 1.0 cm; however, false positives ranged from 1.1 to 5.6 cm. The false negatives had a mean (SD) maxSUV of 2.0 (0.4), whereas the false positives had a mean (SD) maxSUV of 5.6 (3.0). Emphysema was associated with 1.1 higher odds of malignancy, and evidence of granulomatous disease was associated with 0.34 lower odds of benign disease, however, neither was statistically significant (*p* = 0.92 and *p* = 0.31, respectively). Higher maxSUV was significantly associated with increased risk of malignancy (*p* = 8.3 × 10^−3^). Older age and larger size of lesion were borderline associated with increased risk of malignancy (*p* = 0.05 and *p* = 0.07, respectively).

**Conclusion:**

In an area of high endemic granulomatous disease, the PET/CT threshold maxSUV of 2.5 retains a high sensitivity (95.1 %) and positive predictive value (90.6 %) for differentiating benign from malignant pulmonary lesions; however, the specificity (45.5) and negative predictive value (62.5) decrease due to increased false positives. The presence of emphysema and absence of evidence of granulomatous disease increases the probability that a pulmonary lesion is malignant; however, these were not statistically significant.

## Introduction

18-Fluorodeoxyglucose (FDG) positron emission tomography–computed tomography (PET/CT) is an imaging modality that is often used to help differentiate benign from malignant pulmonary lesions [1]. PET/CT has also been shown to improve the accuracy of staging non-small cell lung cancer [2]. The fusion modality PET/CT has gained popularity because of its higher accuracy for evaluation of lesions compared to either PET or CT alone [3, 4]. Malignant lesions have been shown to have elevated expression of the glucose transporter (GLUT-1) and tend to have increased metabolic activity evidenced by increased FDG uptake [5]. However, several benign conditions have also been noted to have increased metabolic activity including infections, granulomatous disease and tuberculosis [6–8]. The threshold maximum standardized uptake value (maxSUV) of 2.5 has been suggested as having reasonable receiver operator characteristics to differentiate benign from malignant pulmonary lesions [9].

We investigate the performance of PET/CT in subjects from Northern California where there is highly endemic granulomatous disease (coccidioidomycosis) [10]. Unlike several prior series investigating the use of PET/CT, all diagnoses were confirmed by histopathology. The accuracy, sensitivity and specificity of the threshold maxSUV value of 2.5 for evaluation of lesions were calculated. Finally we considered other imaging features that may increase the reader accuracy of PET/CT.

## Materials and methods

### Subjects

The study was approved by the Institutional Review Board (IRB) at the University of California, San Francisco Medical Center.

The data are derived from retrospective review of the medical records and imaging of 72 subjects who underwent either fine needle aspiration (FNA) or surgical biopsy of solid solitary pulmonary lesions resulting in a definitive pathologic diagnosis and had a PET/CT study performed for evaluation of the solitary pulmonary lesion in the 2 months prior to the surgical biopsy or FNA. All PET/CT studies were performed between January 2010 and November 2011.

### Imaging technique

All subjects underwent whole body non-contrast enhanced PET/CT, and all scans were acquired using the same PET/CT scanner [General Electric (GE) Discovery 690 (Wisconsin, USA)]. A 64 slice General Electric LightSpeed multi-detector VCT was the Computed Tomography (CT) scanner used in the PET/CT system. The arms were positioned above the subject’s head for CT acquisition, except for subjects with restricted range of movement or shoulder pathology where subject’s arms were positioned at the subject’s sides.

Subjects fasted for at least 6 h prior to the study. CT was acquired from the vertex to the toes without intravenous contrast approximately 60 min following the intravenous administration of 9.0–12.0 mCi (3.33 × 10^2^ to 4.44 × 10^2^ MBq) of FDG, followed by an emission positron emission tomography (PET) scan. PET images were attenuation corrected using the CT transmission data. Axial, coronal and sagittal PET images with and without attenuation correction, and a rotating three-dimensional (3D) maximum intensity projection (MIP) image, were obtained for study interpretation. The non-attenuated corrected images were reviewed because these images are sometimes more accurate for evaluation of pulmonary nodules [11]. Acquired CT and fused PET/CT images were reviewed alongside the uncorrected and corrected PET images. The blood glucose level was less than 160 mg/dL at the time of FDG injection for all subjects.

### Image interpretation

All studies were evaluated by a physician certified in Nuclear Medicine by the American Board of Nuclear Medicine (ABNM). The size (maximum dimension) and maximum standardized uptake value (maxSUV) of each pulmonary nodule was recorded. Evidence of prior granulomatous disease (calcified hilar or mediastinal lymph nodes, calcified pulmonary nodules, liver or splenic granulomas) and presence of emphysema were noted.

### Statistics

Statistics were performed using *R*v2.9.1 statistical software (www.r-project.org). Exact two-sample tests of proportion were used for comparison of two proportions.

## Results

The mean (range) age in the cohort was 69.9 (54–91) years. A total of 72 lesions were identified with a diagnosis based on histopathology. The mean (range) lesion size was 3.0 (0.8–11.0) cm. The mean (range) maxSUV was 10.8 (0.8–36.8). 70.8 % (51/72) of subjects had emphysema and 25.0 % (18/72) had evidence of granulomatous disease (Table 1).

**Table 1:** Study sample characteristics

	Mean (SD)	Range
Age	69.9 (9.0)	54–91
Sex (Male %)	97.2 % (70/72)	–
Size (mm)	30.3 (22.0)	8–110
Maximum SUV	10.8 (7.8)	0.8–36.8
Presence of emphysema % (proportion)	70.8 % (51/72)	–
Evidence of granulomatous disease % (proportion)	25.0 % (18/72)	–

Most (84.7 %) of the lesions with histopathological diagnoses were malignant, and most (96.7 %) of these lesions were primary bronchogenic malignancies. The most common malignancies were adenocarcinoma [47.5 % (29/61)], squamous cell carcinoma [32.8 % (20/61)], unspecified non-small cell lung cancer [11.5 % (7/61)] and metastases [3.3 % (2/61)]. Not all of the lesions that were biopsied were malignant. 15.3 % (11/72) of the biopsied lesions were benign. The most common benign lesions that were biopsied were inflammatory lesions [8.3 % (6/72)], granulomatous lesions (tuberculosis and coccidiodomycosis) [4.2 % (3/72)], non-granulomatous infection [1.4 % (1/72)] and nodular pulmonary amyloidosis [1.4 % (1/72)] (Table 2).

**Table 2:** Histopathological diagnoses present

Pathology	% (Proportion)
Benign	
Inflammatory	8.3 (6/72)
Tuberculosis	2.8 (2/72)
Amyloidosis	1.4 (1/72)
Bronchopneumonia and abscess	1.4 (1/72)
Coccidiodoma	1.4 (1/72)
Malignant	
Adenocarcinoma	40.3 (29/72)
Squamous cell	27.8 (20/72)
Unspecified NSCLC	9.7 (7/72)
Metastases	2.7 (2/72)
Small cell lung cancer	1.4 (1)
Large cell neuroendocrine carcinoma	1.4 (1)
Sarcomatoid carcinoma	1.4 (1)

Using a maxSUV threshold of 2.5, 63/72 biopsied lesions were characterized correctly according to biopsy results giving an accuracy of 87.5 %. The sensitivity and specificity using the maxSUV threshold of 2.5 was 95.1 % (58/61) and 45.5 % (5/11), respectively (Table 3). The three false negative lesions (3/72) had a mean (SD) maxSUV of 2.1 (0.4) and a mean (SD) size of 8.7 (1.2) mm. The false positive lesions (6/72) had a mean (SD) maxSUV of 5.6 (3.0) and a mean (SD) size of 24 (17.2) mm.

**Table 3:** PET/CT maximum SUV threshold of 2.5 performance for characterization of solitary pulmonary nodules

	Accuracy (%)	Sensitivity (%)	Specificity (%)	PPV (%)	NPV (%)
All (*N* = 72)	87.5 (63/72)	95.1 (58/61)	45.5 (5/11)	90.6 (58/64)	62.5 (5/8)
Emphysema (*N* = 51)	84.3 (43/51)	92.9 (39/42)	44.4 (4/9)	88.6 (39/44)	57.1 (4/7)
Granulomatous disease (*N* = 18)	83.3 (15/18)	92.9 (13/14)	50.0 (2/4)	86.7 (13/15)	66.7 (2/3)
Emphysema without granulomatous disease (*N* = 42)	88.1 (37/42)	94.4 (34/36)	50.0 (3/6)	91.9 (34/37)	60.0 (3/5)
Granulomatous disease without emphysema (*N* = 9)	100 (9/9)	100 (8/8)	100 (1/1)	100 (8/8)	100 (1/1)

*PPV* positive predictive value

*NPV* negative predictive value

Emphysema—all subjects with emphysema

Emphysema without granulomatous disease—subjects with only emphysema

Granulomatous disease—all subjects with granulomatous disease

Granulomatous disease without emphysema—subjects with only granulomatous disease

Older individuals were more likely to have malignant lesions, however, this was borderline statistically significant (*p* = 0.05). Larger lesions were more likely to be malignant, however, this was also borderline statistically significant (*p* = 0.07). Higher metabolic activity measured by maxSUV was significantly associated with increased risk of malignancy (*p* = 8.3 × 10^−3^) (Table 4). In the multivariate analysis, the presence of emphysema was associated with an increased risk of malignancy, however, this was not statistically significant (*p* = 0.92). The power of this analysis was limited because most subjects had emphysema. Similarly, evidence of prior granulomatous disease was associated with a decreased risk of malignancy, however, this was not statistically significant (*p* = 0.31).

**Table 4:** Logistic regression evaluating predictors of malignancy

Variable	Odds ratio	95 % CI	*p* value
Univariate			
Age	1.10	(1.00,1.20)	0.05*
Size of lesion (mm)	1.05	(1.00, 1.11)	0.07
MaxSUV	1.47	(1.10, 1.96)	8.3 × 10^−3^*
Presence of emphysema	0.52	(0.10, 2.65)	0.43
Evidence of granulomatous disease	0.52	(0.13, 2.05)	0.35
Multivariate^a^			
Presence of emphysema	1.11	(0.13, 9.19)	0.92
Evidence of granulomatous disease	0.34	(0.04, 2.67)	0.31

* *p* value ≤0.05

^a^Multivariate analyses adjusted for age, size of lesion and maximum SUV

**Fig. 1:**
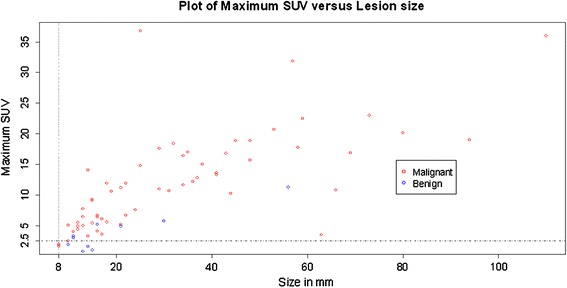
Plot of Maximum SUV versus lesion size

Figure 1 shows a plot of the maxSUV versus size of pulmonary lesion. There was a positive correlation between the size of the lesion and the maxSUV (*r* = 0.71, *p* = 6.2 × 10^−7^) indicating that larger lesions were associated with increased metabolic activity and therefore increased risk of malignancy, however, some of the larger lesions were also benign.

## Discussion

**Fig. 2:**
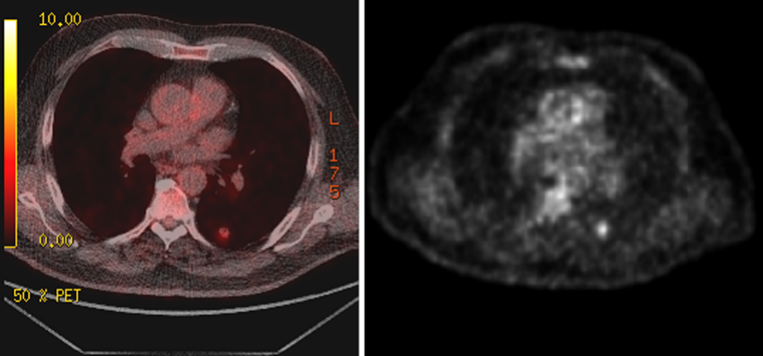
Fused PET/CT image (*left*) and non-attenuated corrected PET image (*right*) demonstrating an intense FDG uptake in a coccidiodoma seen in the left lung

PET/CT remains a useful tool for the evaluation of solid solitary pulmonary lesions. It has been postulated that a solid pulmonary nodule with a maxSUV greater than 2.5 in the right clinical setting should be considered suspicious for malignancy. However, with this maxSUV threshold of 2.5, there are both false positives and false negatives. In our study, we noted far more false positive than false negative results. The increased false positive results were in part related to endemic granulomatous disease. The false negative results were likely related to the small size of the lesion (less than 10 mm). Only one of the six lesions that were less than 10 mm had a maxSUV greater than 2.5. The false positive results were due to infections, including tuberculosis and coccidiodomycosis. These false positive lesions were on average 9.6 mm smaller, but this was not statistically significant (*p* = 0.25), however, the false positive lesions did have significantly lower maxSUV values than the true positive lesions (*p* = 8.0 × 10^−4^). Figure 2 shows a subject with a false positive PET/CT due to a hypermetabolic coccidiodoma.

The accuracy, sensitivity, specificity, negative predictive value and positive predictive value of the maximum SUV threshold of 2.5 in this study were 87.5, 95.1, 45.5, 62.5, and 90.6 %, respectively. The sensitivity and specificity of a test are fixed parameters, which do not change, however, the positive and negative predictive values of a test varies depending on the underlying disease prevalence. The subjects in our study were from Northern California, an area that has a high prevalence of granulomatous disease, which contributed to some of the false positive findings.

Our results are similar to previously published studies. First, we find that bronchogenic carcinoma was the most frequent cause of hypermetabolic solid pulmonary lesions, similar to Ost et al. [8]. The lesion size, age, smoking history and nodule margins are known risk factors for malignancy [8, 12, 13]. In our study, we also find that age (older individuals), and size of lesion (larger lesions) were borderline significant risk factors for nodules being malignant. We also found some evidence that smoking history evidenced by the presence of emphysema was associated with an increased risk of malignancy, however, this was not statistically significant. Li et al. [14] have found that there are increased false positive PET findings in areas with a high prevalence of tuberculosis (TB). Our results also show that there are also increased false positive PET/CT findings in areas with a high prevalence of endemic granulomatous disease.

PET/CT has decreased accuracy in evaluating small pulmonary nodules due to partial volume effect, in particular nodules less than 8 mm [15], and is therefore only recommended by the Fleischner Society for evaluation of pulmonary nodules greater than 8 mm in size or in patients with a history of malignancy [16]. In our study, the false negative results occurred with lesions that were less than 10 mm in size.

This study was done evaluating single time point PET/CTs. Dual time point PET/CT has been proposed as another method of evaluating solitary pulmonary nodules with conflicting evidence. In one study, dual time point PET/CT appeared to be valuable in differentiating benign from malignant solitary pulmonary nodules in areas of high prevalence of granulomatous disease [7]. However, other studies have shown that dual time point PET/CT has similar accuracy compared to single time point PET/CT [17, 18], and Chen et al. [6] conclude that dual time point PET/CT was not useful, especially in areas with a high prevalence of granulomatous disease [6]. These discrepant results may be due to differences in technique. The maximum FDG uptake for lung cancers occurs at around 5 h (300 min) after injection, and therefore dual time point PET/CT imaging may in some cases appear no better than single time point PET/CT imaging if the second imaging is done 120–180 min after injection of the radiotracer [19].

There are a few limitations to our study. First, the study was retrospective in nature and therefore subject to case selection bias. Another limitation of our study was that the lesions that did not go on to excision/biopsy were excluded from the analysis. Although this is a limitation, it is also a strength of this study. Histopathologic proof was used to determine the diagnosis of each of the pulmonary lesions. This diminishes uncertainty and potential misdiagnoses that can occur when criteria such as lack of interval change on imaging studies are used for indicating the true diagnoses of the pulmonary lesions. Most of the subjects in the study were male, which may limit generalizability of the results. Finally, we use the common maximum SUV threshold value of 2.5, which has been challenged in the literature as being too high and not specific enough to rule in underlying malignancy [20, 21]. While there remains significant debate around this threshold, at our institution, all lesions that are not excised/biopsied are followed for at least 2 years to document stability.

Evaluation of solitary pulmonary nodules remains a diagnostic challenge. Over 150,000 patients annually present to their physicians with the diagnostic dilemma of a solitary pulmonary nodule found either on chest X-ray or chest CT [22]. Lung cancer has the highest incidence and highest mortality in the United States [23], and early detection of lung cancers greatly improves survival [23]. This study substantiates the use of PET/CT for characterization of solid pulmonary lesions in subjects from areas with endemic granulomatous disease; however, there are increased false positive findings due to granulomatous disease in this setting.

## Summary

In an area of high endemic granulomatous disease, the PET/CT threshold maxSUV of 2.5 retains a high sensitivity (95.1 %) and positive predictive value (90.6 %) for differentiating benign from malignant pulmonary lesions; however, the specificity (45.5 %) and negative predictive value (62.5 %) decrease due to increased false positives. This suggests that further work needs to be done to assess how the performance of PET/CT can be improved to evaluate solid solitary pulmonary lesions in patients from areas with endemic granulomatous disease. The presence of emphysema and absence of evidence of granulomatous disease increases the probability that a pulmonary nodule is malignant; however, these were not statistically significant (*p* = 0.92 and *p* = 0.31, respectively).
